# Associations of the gut microbiome with hepatic adiposity in the Multiethnic Cohort Adiposity Phenotype Study

**DOI:** 10.1080/19490976.2021.1965463

**Published:** 2021-09-07

**Authors:** Meredith A. J. Hullar, Isaac C. Jenkins, Timothy W. Randolph, Keith R. Curtis, Kristine R. Monroe, Thomas Ernst, John A. Shepherd, Daniel O. Stram, Iona Cheng, Bruce S. Kristal, Lynne R. Wilkens, Adrian Franke, Loic Le Marchand, Unhee Lim, Johanna W. Lampe

**Affiliations:** a Public Health Sciences, Fred Hutchinson Cancer Research Center, Seattle, Washington, U.S.A; b Clinical Research Division, Fred Hutchinson Cancer Research Center, Seattle, Washington, U.S.A; c Preventive Medicine, Keck School Of Medicine, University Of Southern California, Los Angeles, California, U.S.A; d John A. Burns School Of Medicine, University Of Hawaii, Honolulu, Hawaii, U.S.A; e University Of Hawaii Cancer Center, University Of Hawaii, Honolulu, Hawaii, U.S.A; f Keck School Of Medicine, University Of Southern California, Los Angeles, California, U.S.A; g School Of Medicine, University Of California San Francisco, San Francisco, California, U.S.A; h Department Of Medicine, Brigham And Women’s Hospital And Harvard Medical School, Boston, Massachusetts, U.S.A

**Keywords:** nonalcoholic fatty liver disease (NAFLD), inflammation, hepatocellular carcinoma, chronic liver disease, ethnic groups, genetics, diabetes, metabolic syndrome

## Abstract

Nonalcoholic fatty liver disease (NAFLD) is a risk factor for liver cancer and prevalence varies by ethnicity. Along with genetic and lifestyle factors, the gut microbiome (GM) may contribute to NAFLD and its progression to advanced liver disease. Our cross-sectional analysis assessed the association of the GM with hepatic adiposity among African American, Japanese American, White, Latino, and Native Hawaiian participants in the Multiethnic Cohort. We used MRI to measure liver fat and determine nonalcoholic fatty liver disease (NAFLD) status (n = 511 cases) in 1,544 participants, aged 60–77 years, with 12–53% overall adiposity (BMI of 17.8–46.2 kg/m^2^). The GM was measured by 16S rRNA gene sequencing and, on a subset, by metagenomic sequencing. Alpha diversity was lower overall with NAFLD and in certain ethnicities (African Americans, Whites, and Latinos). In models regressing genus on NAFLD status, 62 of 149 genera (40%) exhibited a significant interaction between NAFLD and ethnicity stratified analysis found 69 genera significantly associated with NAFLD in at least one ethnic group. No single genus was significantly associated with NAFLD across all ethnicities. In contrast, the same bacterial metabolic pathways were over-represented in participants with NAFLD regardless of ethnicity. Imputed secondary bile acid and carbohydrate pathways were associated with NAFLD, the latter of which was corroborated by metagenomics, although different genera in different ethnicities were associated with these pathways. Overall, we found that NAFLD was associated with altered bacterial composition and metabolism, and that bacterial endotoxin, assessed by plasma lipopolysaccharide binding protein (LBP), may mediate liver fat-associated systemic inflammation in a manner that seems to vary by ethnicity.

## Introduction

Nonalcoholic fatty liver disease (NAFLD) is estimated to affect >30% of the US population and is closely associated with elevated risk for cardiovascular disease, metabolic syndrome, type 2 diabetes (T2D), and overall malignancy. Metabolic disorders, including NAFLD – hepatic manifestations of obesity – now contribute more to the risk of hepatocellular carcinoma (HCC) than any other etiologies for this cancer in the US.^1^ Multiple risk factors for NAFLD include ethnicity, genetics, diet, and the gut microbiome (GM) and its metabolites.^2^ Setiawan et al.^3^ found that NAFLD was the most common etiology for chronic liver disease (CLD) in all five ethnic groups in the Multiethnic Cohort Study (MEC); however, the proportion of NAFLD-associated CLD varied across ethnic groups. Obesity, especially visceral adiposity which varies across ethnicity, was associated with NAFLD.^4^ Diet and diet quality also varying across ethnic groups, was associated with NAFLD^5,6^ and may play a role in ethnic differences in risk of CLD, which have also been associated with different patterns of dietary intake.^7,8^

The gut-liver axis, whereby the liver is exposed to gut microbial metabolites and endotoxins (i.e., gram-negative bacterial cell wall material), may promote inflammation in the development of NAFLD. GM composition and microbial metabolic pathways,^9–11^ may influence host metabolism, oxidative stress, and systemic and liver inflammation. Previous studies have identified reduced bacterial diversity with NAFLD but have not identified consistently specific bacterial genera associated with NAFLD, although this discrepancy may be due to small sample sizes and heterogeneous study populations.^9,10,12–15^ This has limited the potential to develop biomarkers of ectopic fat related to disease risk stratified along ethnicity.^4^ Despite evidence that NAFLD varies across ethnicities,^4,16^ the variation in how the GM is associated with NAFLD has not been investigated across ethnic groups.

Our aim was to examine, in a cross-sectional subset of the Multiethnic Cohort, the associations between NAFLD and GM composition and bacterial metabolic pathways. This analysis took advantage of a relatively large sample of the cohort’s five ethnic groups and the state-of-the-art measurements of liver at and NAFLD using MRI It affords the opportunity to measure the association between the microbiome structure and function that may link pathophysiologic pathways to potential clinical relevance^17^in treating the racial/ethnic disparity in the severity of NAFLD.

## Materials/subjects and methods

### Study population

The MEC study has followed 215,000 Hawaii and Los Angeles residents of African American, Japanese American, white, Latino, and Native Hawaiian origin since 1993–1996.^18^ The Multiethnic Cohort – Adiposity Phenotype Study (MEC-APS) examined 1,861 healthy MEC participants, including men and postmenopausal women aged 60–77 years, of the five main MEC ethnic groups.^4^ The recruitment was stratified by sex, ethnicity and six body mass index (BMI) categories to balance distribution across a wide range of BMI in each sex-ethnic group. Between 2013 and 2016, participants visited the two affiliated clinical centers in Honolulu, Hawaii, or Los Angeles, California, to undergo a whole-body dual-energy X-ray absorptiometry (DXA) and abdominal magnetic resonance imaging (MRI) scan, anthropometric and resting metabolism measurements, fasting blood and stool sample collections, and repeat administration of the MEC food frequency questionnaire (FFQ) covering over 180 food items including ethnic-specific foods.^5,19^ Usual dietary intake and the Healthy Eating Index-2010^20^(HEI-2010; as a measure of overall diet quality) over the past year were estimated from the FFQ.

As detailed in a previous report,^4^ individuals were excluded for current or recent (<2 years) smoking, amputation or implants, claustrophobia, insulin or thyroid medication, dialysis, and serious health conditions such as chronic viral hepatitis and dialysis. Also, participation was deferred for those with recent history within 6 months of chemotherapy or radiation therapy of the abdomen, antibiotic used, substantial weight change (>20 pounds) or colonoscopy as well as vaccination within 1 month. We also limited the current analysis to nill to moderate alcohol drinkers (men <30 g/day, women <20 g.day based on FFQ). Our study was based on 1,544 of the 1,861 MEC-APS participants after removing 74 that were missing microbiome data, 62 missing liver fat data mostly due to motion artifacts, and 181 who reported high alcohol use. For adjustment of total adiposity, we also removed 15 participants without valid DXA measure which resulted in 1,529 participants in the adjusted analysis including Latinos (n = 325) and African Americans (n = 256) recruited in Los Angeles and Native Hawaiian (n = 246), all whites (n = 316), and Japanese Americans (n = 400), as well as one African American recruited in Hawaii.

The study protocol conformed to the ethical guidelines of the 1975 Declaration of Helsinki and Institutional Review Board reviews were conducted by participating institutions. Signed informed consent was obtained from all study participants.

### Assessment of liver fat and NAFLD

We measured percent liver fat using abdominal MRI and total body fatness using DXA as previously described^4^ with a method that has shown high accuracy compared to liver biopsy.^21^ NAFLD was defined as percent liver fat of >5.5%.^22^

### Blood biomarkers

Venous blood (40 mL) was collected after an overnight fast (>8 hours), processed in the MEC laboratories in Hawaii and Los Angeles to components (plasma, buffy coat, serum), and stored at −80°C until shipment for biomarker assays at the UH Cancer Center Analytical Biochemistry Shared Resource laboratory (directed by Dr. Franke). Samples were arranged into batches so that each batch included approximately equal numbers of men and women of each ethnic group and ~10% blind QC duplicates. Plasma lipopolysaccharide binding protein (LBP) was analyzed using a commercial ELISA kit (Cell Sciences, CKH113; coefficient of variation (CV): 0.7%; intraclass correlation coefficient (ICC) 80%). Serum high-sensitivity C-reactive protein (CRP), a measure of systemic inflammation and alanine amino transferase (ALT), a plasma marker of liver dysfunction, were measured as previously reported.^23^ Our assays for CRP and ALT had a CV of 13.8% and 4.4% and ICCs of 88% and 82%, respectively.

### Microbiome Analysis

#### Sample collection

Stool samples were collected at home using a collection tube containing 5 mL RNAlater (Fisher Scientific) and sterile 5 mm glass beads (Ambion) to facilitate sample dispersion in RNAlater.^24^ Participants kept their samples in their freezers and brought them to the study clinic. At sample collection, participants filled out a questionnaire to provide collection time, special diets, consumption of probiotic foods in the past year, and whether participants were treated with an oral, injection, or IV form of antibiotics in the past year, and if so, how recently.

#### Sample processing

Stool samples were stored in RNAlater at −80°C at study centers and shipped in bulk on dry ice to Fred Hutchinson Cancer Research Center (Fred Hutch). Stool samples were thawed and homogenized, and genomic DNA was extracted.^24^ Briefly, to optimize bacterial genomic DNA extraction, we did bead beating at 45s (2x) each with samples placed on ice in between. Quality control samples, duplicate participant samples, and processing blanks were used to assess variation in library preparation and sequencing batches.^25^

For paired-end sequencing of the V1–V3 region of the 16S rRNA gene, the 27 F mod forward PCR primer sequence was 5°-AGRGTTNGATCMTGGCTYAG-3°. The 519 R reverse PCR primer sequence was 5°-GTNTTACNGCGGCKGCTG-3°. Three PCR (20 µl; 20 ng genomic DNA) reactions were performed using the HotStarTaq Plus Master Mix Kit (QIAGEN) under the following conditions: 94°C for 3 minutes, followed by 28 cycles of 94°C for 30 seconds, 53°C for 40 seconds, and 72°C for 1 minute, after which a final elongation step at 72°C for 5 minutes was performed. After amplification, quality of the PCR products were checked in 2% agarose gel. The three PCR products were pooled together in equal proportions based on their molecular weight and DNA concentrations. Paired-end sequencing performed at Molecular Diagnostics, LLP (Shallowater, TX) on the MiSeq using MiSeq Reagent Kit v3 following the manufacturer’s guidelines to obtain 2 × 300 bp paired-end reads (Illumina, San Diego, CA). On a subset of samples chosen from the NAFLD vs non-NAFLD groups from each ethnicity (n = 30; six from each ethnicity), we performed whole-genome shotgun sequencing on an Illumina HiSeq generating 2×150bp paired-end reads. FastQ files were exported and securely transferred (BaseSpace, Illumina) to Fred Hutch for bioinformatic analysis.

#### Microbiome bioinformatic data processing

To classify bacterial taxonomy, sequences were processed using QIIME v.1.8^26^ as previously described.^25^ The filtering strategy for operational taxonomic units (OTUs) included parameters in QIIME to exclude low abundant sequences, singletons, and chimeras. Briefly, the OTU table was filtered using the QIIME script filter_OTUs_from_OTU_table.py with – min_count_fraction set to 0.00005. Additional OTU entries were filtered out if they were detected as chimeras using QIIME’s identify_chimeric_seqs.py script with method blast_fragments. Final filtering excluded genera which appeared in <10% of the subjects.^27^ Bacterial functional genes were imputed from 16S rRNA genes counts that were adjusted for batch (see below).^27^

Sequence reads were processed for bioinformatic analysis of the metagenomes (n = 30) with the KneadData v 0.5.1 quality control pipeline, which uses Trimmomatic (version 0.36), BMTagger filtering, and decontamination algorithms to remove low-quality read bases and host (human) reads, respectively.^28^ Trimmomatic was run with parameters MAXINFO:80:0.5 and MINLEN:50. Functional profiling was performed using HUMAnN2 version 0.11.2^29^ with reads de-paired and implementing Diamond^30^ to map reads against UniRef90.^31^ Sequences per gene family were counted, normalized for length and alignment quality, and linked to pathways using MetaCyc.^32^ Data matrices of the abundance of genes, gene families, and genes in metabolic pathways were generated. We quantified the abundance of the UniRef90 gene families and gene names for secondary bile acids (Supplemental Table 2) and carbohydrate metabolism (Table 3)^33^ by summing the normalized counts of all genes that mapped to these pathways.

Enzymes in carbohydrate metabolism were identified using the CAZy database (31 July 2019 ver., URL http://bcb.unl.edu/dbCAN2/download/Databases/CAZyDB.07312019.fa). A DIAMOND (version 0.9.22) database was generated from the FASTA file and used to align metagenomics reads, where hits were retained if the e-value <10^−20^. Data for statistical analysis were filtered for abundance (cells with less than 46 counts which represents the 25th percentile of non-zero counts were placed to zero) and prevalence (variables that had greater than or equal to 75% zero counts were removed). All sequences follow MIMARKS standard^34^ and are publicly available in the Sequence Read Archive (http://www.ncbi.nlm.nih.gov/sra/.)

On unrarefied count data, we applied ComBat-seq^35^ designed to remove unwanted (non-biological) variation, while preserving potentially relevant variation in sequence count data. This was applied by including, for each sample, information on sequencing batch along with each participant’s sex and ethnicity. An additional set of quality-control samples run in each batch were also used in this process. The resulting adjusted counts were used in all subsequent statistical analyses.

### Statistical Analysis

We examined the association between the GM and NAFLD among the 1,529 MEC-APS participants.^35,36^ Results from each significance test are reported as a “q” value and statistical significance was defined by an FDR cutoff less than 0.05.^37^ Analyses were performed using R Version 4.0.2.

#### Participant characteristics

**Figure 1.**
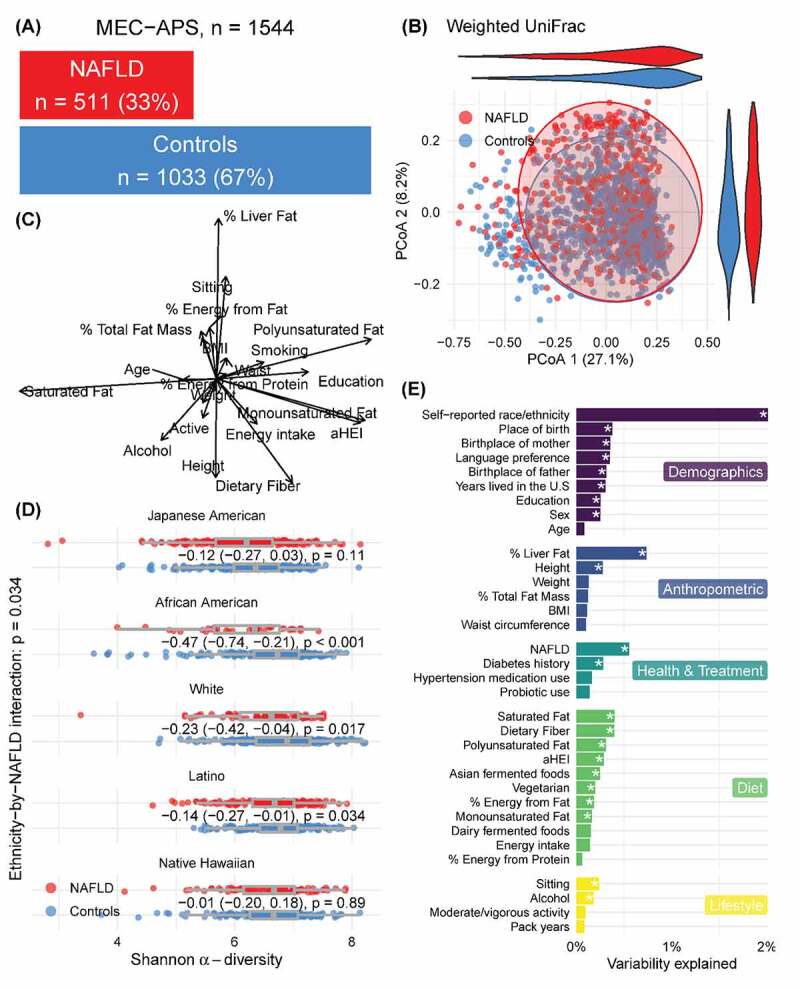
Overview of the microbiome in NAFLD in the MEC-APS study. 1A.) Distribution of individuals with and without NAFLD. 1B.) Variation in the microbiome between individuals with and without NAFLD illustrated by a PCOA of weighted Unifrac metric. 1 C.) Vector overlay of the demographic, anthropometric, health, diet, and lifestyle variables. 1D.) Alpha diversity in subjects with and without NAFLD by ethnicity and 1E.) Variation in the microbiome explained by the demographic anthropometric, health, diet, and lifestyle variables using perMANOVA analysis

Demographic, anthropometric, health, lifestyle, and dietary measures were examined overall and by NAFLD status (Table 1, Figure 1e, and Supplemental Figures 5E–9E). These were assessed using t-tests for continuous variables and chi-squared tests for categorical variables as were the subset of participants for which we did metagenomic sequencing (Supplemental Table 4).

**Table 1.** MEC-APS participant characteristics by NAFLD status (liver fat>5.5%)

	**NAFLD status**			
	**No** **(N = 1033)**	**Yes** **(N = 511)**	***P*^a^**	**Total** **(N = 1544)**
**Demographics**				
Ethnicity, n (%)			<0.001^b^	
Japanese American	222 (21)	178 (35)		400 (26)
African American	219 (21)	38 (7)		257 (17)
White	249 (24)	67 (13)		316 (20)
Latino	182 (18)	143 (28)		325 (21)
Native Hawaiian	161 (16)	85 (17)		246 (16)
Sex – Female, n (%)	533 (52)	267 (52)	0.809^b^	801 (52)
Age (years)	69.3 (2.8)	69.0 (2.7)	0.03	69.2 (2.7)
Education (years)	15.0 (2.6)	14.4 (2.9)	<0.001	14.8 (2.7)
Born in US, n (%)	888 (86)	416 (82)	0.022	1304 (85)
Mother born in US, n (%)	845 (82)	395 (78)	0.042	1240 (81)
Father born in US, n (%)	806 (78)	358 (70)	< 0.001	1164 (76)
Lived in US 26+ years, n (%)	952 (93)	458 (90)	0.087	1410 (92)
Primary language English, n (%)	988 (96)	468 (92)	0.001	1456 (94)
**Anthropometrics**				
% Liver Fat	3.2 (1.1)	10.7 (4.8)	< 0.001	5.6 (4.6)
Height (cm)	165 (10)	163 (10)	< 0.001	165 (10)
Weight (kg)	73 (15)	81 (16)	< 0.001	76 (16)
Total Fat Mass^c^ (kg)	32.9 (8.2)	35.1 (6.7)	<0.001	33.7 (7.8)
BMI (kg/m^2^)	26.5 (4.5)	30 (4.5)	< 0.001	28 (5.0)
Waist circumference (cm)	94 (12.5)	101.9 (10.9)	< 0.001	96.5 (12.5)
**Health and Treatment**				
Diabetes history, n (%)	114 (11)	132 (26)	< 0.001	246 (16)
Hypertension medication use, n (%)	375 (36)	255 (50)	< 0.001	630 (41)
Probiotic use, n (%)	104 (10)	42 (8)	0.243	146 (9)
**Diet**				
Energy Intake (kcal/day)	1805 (834)	1839 (892)	0.46	1816 (854)
% Energy from Fat	33.5 (6.6)	34.7 (6.0)	< 0.001	33.9 (6.2)
% Energy from Protein	16 (3)	17 (3)	0.008	17 (3)
Dietary Fiber (g/1000 kcal/day)	13.3 (4.4)	11.9 (3.9)	<0.001	12.9 (4.3)
Saturated Fat (% of total fat)	32.9 (4.8)	33.8 (4.2)	<0.001	33.2 (4.7)
Polyunsaturated Fat (% of total fat)	25.2 (4.1)	24.1 (3.7)	<0.001	24.8 (4.0)
Monounsaturated Fat (% of total fat)	41.9 (2.8)	42.0 (2.5)	0.54	42.0 (2.7)
Healthy Eating Index 2010 (aHEI-2010)	69.7 (10.1)	66.3 (9.7)	< 0.001	68.6 (10.1)
Asian fermented foods, n (%)	250 (24)	167 (33)	< 0.001	417 (27)
Dairy fermented foods, n (%)	463 (45)	227 (44)	0.882	690 (45)
Vegetarian diet, n (%)	28 (3)	11 (2)	0.511	39 (3)
**Lifestyle**				
Sitting (hours/day)	8.0 (3.4)	8.4 (3.5)	0.012	8.1 (3.4)
Alcohol (g/day)	4.5 (6.7)	3.1 (5.9)	<0.001	4.0 (6.5)
Moderate/vigorous activity (hours/day)	1.7 (1.5)	1.3 (1.2)	< 0.001	1.5 (1.4)
Smoking (pack years)	4 (9)	5 (10.5)	0.049	0.08 4 (9.5)

Continuous variables reported as mean (standard deviation). Categorical variables reported as n (%).

^a^t-test used, unless noted

^b^Pearson’s Chi-squared test

^c^Total fat mass was missing in 15 participants (12 No NAFLD and 3 Yes NAFLD)

#### Alpha and beta diversity

Linear regression was used to model Shannon alpha diversity by NAFLD status and its interaction with ethnicity, adjusted for sex and total adiposity (from DXA) and in similar models stratified by ethnic groups. To quantify the extent to which various demographics, anthropometric, health, diet, and lifestyle measures were associated with the overall gut microbiota composition, we implemented perMANOVA^38^ using weighted UniFrac distances between pairs of taxon abundance vectors (Supplemental Table 5).^39^

#### Phyla and Genera

Beta-binomial regression models were fit, marginally, for each genus and phylum using the R package ‘corncob.’^40^ The observed count of each taxon and the sample’s sequencing depth were used to model the population proportion of each taxon relative to covariates. The beta component models the probability parameter of the binomial distribution to better account for the overdispersion and contains mean and overdispersion components for each taxon. For genera present in at least 5% of the entire participants, we first modeled each taxon by including a NAFLD-ethnicity interaction term, adjusted by sex and percent total fat for both mean and overdispersion components. Since this interaction was significant in 62 out of 149 genera (Supplementary Table 1B), we concluded there is strong evidence for ethnic-specific associations between NAFLD status and taxon abundance and refitted these models stratified by each of the five ethnic groups. In analyses stratified by ethnicity, we only modeled those genera present in at least 25% of each group. We used a likelihood ratio test to assess differences in mean proportions by NAFLD status.

#### The association of microbial metabolic pathways with liver fat

Linear regression was used to model percent liver fat, as the dependent variable, by piCRUST-imputed metabolic pathways.^27^ Pathway counts were adjusted for batch using ComBat-seq and each sample was normalized using the centered-log ratio (CLR).^41^ CLR values were standardized (mean 0 and standard deviation 1) for comparison of pathway coefficients across models. Models were adjusted for total adiposity, sex, and self-reported ethnicity. We fit additional models stratified by sex and ethnicity adjusted only for total adiposity.

In a subset of 27 samples that were assessed using metagenomic sequencing, we tested whether the relative abundance of sequenced genes in six metagenomic pathways differed by NAFLD status using a Wilcoxon rank-sum test. We similarly tested 146 glucosyl hydrolase enzymes.

#### Mediation analysis

Mediation analysis was used to assess whether the association between percent liver fat and a marker of systemic inflammation (CRP) was mediated by LBP. For this analysis, we used 1,326 participants having non-missing, non-zero measures of CRP. In models stratified by ethnicity, we estimated direct and indirect effects by regressing log(CRP) on percent liver fat, with and without LBP, and regressing of LBP onto percent liver fat. Bootstrapped confidence intervals were used to evaluate the direct, indirect, and mediating effects.^42,43^ All models were adjusted for sex and percent total fat mass.

## Results

### Participant characteristics by NAFLD status

As reported previously,^4^ the prevalence of NAFLD varied widely by ethnicity, especially higher in Japanese Americans and lower in African Americans than in other groups (Table 1). As expected, NAFLD cases, compared to non-cases, had higher mean levels of percent liver fat, total fat mass, BMI, waist circumference, higher prevalence of diabetes history and hypertension medication use. NAFLD cases and non-cases had similar intake of probiotics and total energy, but cases had higher percent energy from fat, protein, and saturated fats and lower levels of energy-adjusted dietary fiber and overall diet quality, determined with a HEI-2015 score. A sedentary lifestyle and smoking were significantly higher in participants with NAFLD whereas alcohol intake and activity were higher in participants without NAFLD. Mean plasma LBP, CRP, and ALT concentrations were greater in the NAFLD group overall and within each ethnic group, although significance varied by ethnicity (Table 2).

**Table 2.** **Plasma Concentrations of LBP, CRP and ALT** in MEC-APS participants by NAFLD status, overall and stratified by ethnicity

	NAFLD status			
	No	Yes	Total	*P*
**All**	**N = 1033**	**N = 511**	**N = 1544**	
LBP	22.4 (8.0)	24.5 (8.3)	22.2 (8.5)	< 0.001
CRP	1.60 (2.2)	2.25 (2.8)	1.82 (2.4)	< 0.001
ALT	19.6 (11.0)	26.1 (14.3)	21.7 (12.6)	< 0.001
**Japanese American**	**N = 222**	**N = 178**	**N = 400**	***q*^a^**
LBP	21.2 (7.5)	22.6 (8.0)	21.8 (7.8)	0.865
CRP	0.8 (1.5)	1.4 (1.8)	1.1 (1.6)	0.101
ALT	21.0 (9.6)	28.9 (16.0)	24.5 (13.4)	<0.001
**African American**	**N = 219**	**N = 38**	**N = 257**	
LBP	22.9 (9.3)	25.7 (9.9)	23.3 (9.4)	0.163
CRP	2.5 (2.9)	4.1 (5.1)	2.7 (3.4)	0.051
ALT	18.9 (12.9)	21.4 (9.3)	19.3 (12.5)	0.295
**White**	**N = 249**	**N = 67**	**N = 316**	
LBP	22.2 (8.4)	25.1 (7.5)	22.8 (8.3)	0.396
CRP	1.4 (1.7)	2.9 (3.2)	1.7 (2.2)	<0.001
ALT	19.1 (9.0)	27.3 (15.2)	20.8 (11.1)	<0.001
**Latino**	**N = 182**	**N = 143**	**N = 325**	
LBP	22.4 (7.7)	25.2 (8.4)	23.6 (8.2)	0.049
CRP	2.0 (2.3)	2.7 (2.6)	2.3 (2.5)	0.132
ALT	19.1 (10.1)	25.0 (13.6)	21.7 (12.1)	<0.001
**Native Hawaiian**	**N = 161**	**N = 85**	**N = 246**	
LBP	21.7 (7.5)	24.2 (7.9)	22.6 (7.7)	0.227
CRP	1.3 (1.9)	2.0 (2.5)	1.5 (2.2)	0.191
ALT	19.8 (13.3)	23.5 (11.4)	21.1 (12.7)	0.024

Variables reported as mean (standard deviation).

^a^t-test used

*Models were adjusted for ethnicity, sex, total fat mass, and batch.

Abbreviations and Units: LBP, lipopolysaccharide binding protein (mg/mL); CRP, C-reactive protein (mg/L) and ALT, alanine aminotransferase (U/L).

### Gut microbiome

Microbiome sequencing averaged 33,422 16S rRNA gene sequences per sample with an average length of 499 bp. We identified 10 phyla, 152 genera, and 1,311 OTUs. We sampled metagenomes in a subset of participants (n = 27) which averaged 20.3 M reads ± 2.8 M reads per sample before quality filtering and 20.2 M reads ± 2.9 M reads afterward, with a mean of 0.5% of the reads removed including human and poor quality reads. Sequence length was 148 bp ± 14 bp before QC, and 143 bp ± 19 bp after QC. Three of the original 30 samples were removed from the metagenomic analysis due to participant’s high alcohol consumption leaving a total of 27 samples of which 13 were in the non-NAFLD category and 14 were in the NAFLD category and mapping rate of high-quality reads was similar across ethnic groups (Supplemental Table 6).

We modeled the association of alpha diversity (Shannon Index) with NAFLD in the entire sample with adjustment for sex, total fat mass, ethnicity, and the interaction of NAFLD with ethnicity. Alpha diversity was inversely associated with NAFLD status overall (*p* = 0.034). There was a significant interaction of ethnicity and NAFLD and in stratified analysis among African Americans (*p* < 0.001), whites (*p* = 0.017) and Latinos (*p* = 0.034) (Figure 1d; Supplemental Figures 5D–9D).

We tested the association of overall gut microbial composition (beta diversity) with NAFLD status using perMANOVA^38^ with respect to weighted UniFrac,^39^ including terms for sex, total fat mass, ethnicity, and the interaction of NAFLD with ethnicity. We observed a significant ethnicity−by−NAFLD interaction (*p* = 0.014) which explained 0.44% of the variability. Microbial beta diversity was associated with NAFLD among all participants (q < 0.05) (Figure 1b and 1e). Self-reported ethnicity (2%), percent liver fat (0.74%), NAFLD (0.55%), and dietary fiber (0.4%), and saturated fat (0.04%) were among the variables that explained the most the overall variation in the microbiome (Figure 1e). In ethnicity stratified results (Supplemental Figures 5–9), NAFLD and % liverfat explained a significant but small amount of variation in the overall microbiome composition (Supplemental Figures 5–9 A-F).

Over all participants, at the phylum-level, we observed associations with seven out of 10 phyla and 62 out of 149 genera (Supplemental Table 1A and 1B). Fusobacteria (*p* < 0.0001), Bacteroidetes (*p* < 0.0001), and Proteobacteria (*p* < 0.0001) were positively associated with NAFLD. At the genus level, 14 genera were positively associated with NAFLD (all q < 0.01) and 48 genera (all q < 0.01) were inversely associated with NAFLD (Supplemental Table 1B).

**Figure 2.**
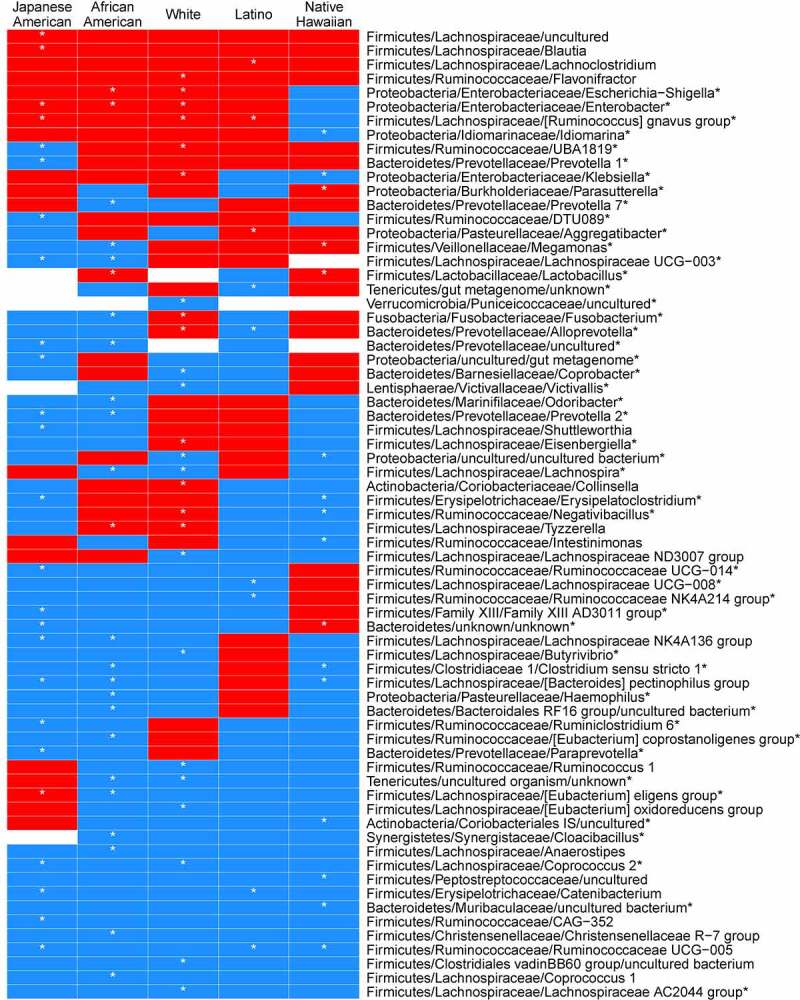
Heatmap of regression coefficients for association with NAFLD resulting from beta-binomial regression models of genera counts stratified by self-report ethnicity. Genera significantly enriched or depleted in individuals with NAFLD are shown. Coefficients are adjusted for sex, and total fat mass. Red = positive, blue = negative

We observed a significant interaction between NAFLD status and ethnicity in phyla (Supplemental Figure 1). Proteobacteria were positively associated with African American subjects with NALFD and Actinobacteria were positively associated with white subjects with NAFLD. Fusobacteria was inversely associated with NAFLD in Blacks and positively associated in whites. Tenericutes were inversely associated with NAFLD in African Americans, whites, and Latinos. Firmicutes and Synergistetes were inversely associated with NAFLD in African Americans and whites. The prevalence and abundance of genera (Supplemental Table 1B; Supplemental Figures 5–9). Genera were significantly positively associated with liver fat in at least one ethnic group (as summarized in Figure 2; Supplemental Table 1B), although only *R. gnavus* group (Japanese Americans, whites, and Latinos) and *Enterobacter* (Japanese Americans, African Americans, and whites)^44^ were found in common across three ethnicities. *Megamonas* was enriched in Hawaiians and depleted in African Americans with NAFLD. *Alloprevotella* was increased in whites but decreased in Latinos, and *Klebsiella* was enriched in whites and decreased in Native Hawaiians. Christensenellaceae was negatively associated with African American subjects with NAFLD. Among those genera that differed significantly in abundance by NAFLD status within a single ethnic group, *Aggregatibacter*^45–47^ and *Lachnoclostridium* were enriched in Latinos with NAFLD, *Blautia* was enriched in Japanese Americans, and *Parasutterella* was enriched in Native Hawaiians. The prevalence and abundance of *Fusobacterium* varied across ethnic groups (Supplemental Table 1B and Supplemental Figure 4).

Several genera were inversely associated with NAFLD although there were some divergent patterns for genera within families across ethnicities (Figure 2; Supplemental Table 1B). We found that among the genera in the Lachnospiraceae family, *Coprococcus-2* was decreased in Japanese Americans and whites whereas *Lachnospira* was decreased African American and whites, and *Lachnospiraceae UCG-003* was decreased in Japanese Americans and African Americans with NAFLD. In the Ruminococcaceae, some genera were unique to an ethnic group: five unique genera only in Japanese Americans, two genera unique to Native Hawaiians and one genera each in Latinos, African Americans, and whites. In the Bacteroidetes, *Prevotella-1* and *Prevotella-7* were decreased in Japanese American and African American participants, respectively, and an uncultured Prevotellaceae was decreased in both. *Coprobacter* was decreased in whites, an uncultured genera in the Muribaculaceae in whites, and *Odoribacter* in African Americans. The most common genera that were significantly inversely associated with NAFLD were the *Bacteroides pectinophilus* group in Japanese Americans, African Americans, and Native Hawaiians and *Ruminococcaceae UCG-005* in Japanese Americans, Latinos, and Native Hawaiians. In the Family Erysipelotrichaceae, *Catenibacterium* was decreased in Japanese American and Latino participants with NAFLD whereas *Erysipelatoclostridium* was decreased in Native Hawaiians and Japanese Americans.

ALT, a measure of liver damage, was associated with genera in a beta binomial regression stratified by ethnicity and adjusted for sex and total adiposity (Supplemental Table 7). *R. gnavus* was significantly positively associated with ALT in three ethnicities: Japanese Americans, whites, and Latinos. *Klebsiella* was significantly positively associated with ALT in whites and Native Hawaiians. *Megamonas* was inversely associated with ALT in African Americans and positively associated in Native Hawaiians. Several single genera in the Lachnospriaceae, Ruminococcaeae, and Christensenellaceae were negatively associated with ALT in a specfic ethnicity.

**Figure 3.**
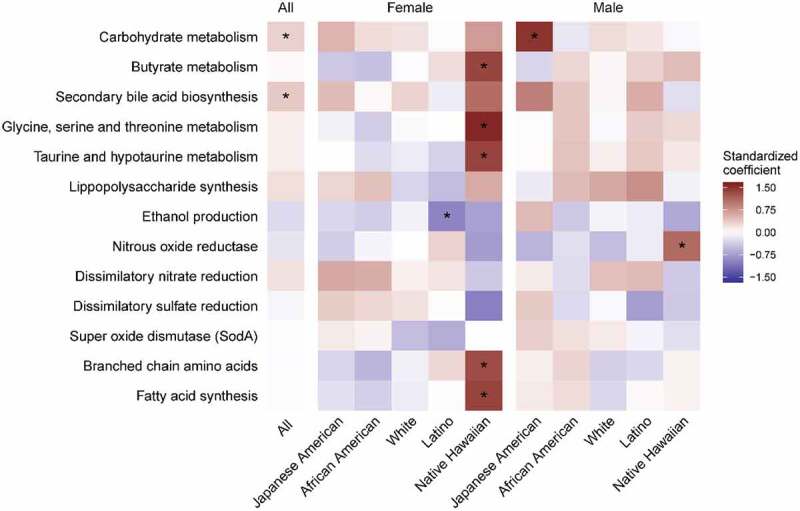
Heatmap of the association between imputed microbial functional pathways with percent liver fat by sex and ethnicity in the MEC-APS (n = 1529). The colors represent the magnitude of the pathway standardized coefficient in the linear model of the natural log of percent liver fat on each pathway, adjusted for sex, ethnicity, total fat mass, and sequencing batch. The gene counts were inferred from piCRUST and pathways summarized by summing all counts in the pathway and then normalized for the total number of sequences per sample, which were then standardized for the model. Asterisks indicate a BH corrected *p* value less than 0.1. (Also see Supplemental Table 5.)

Microbial metabolic pathways were associated with liver fat (Figure 3; Supplemental Table 5, Supplemental Figure 2). Among all participants, we found a positive association of liver fat with imputed bacterial functional genes involved in secondary bile acid metabolism and carbohydrate metabolism. Japanese American males showed a positive association with carbohydrate metabolism and Native Hawaiian females showed a positive association with secondary bile acids metabolism (conjugate metabolism of taurine and C1 amino acids) and other pathways associated with branched chain amino acids and fatty acid synthesis.^48,49^

Metagenomic sequencing of functional genes on a subset of participants (n = 27; Supplemental Table 4) showed that microbial carbohydrate enzymes involved in the degradation of hemicellulose was significantly higher in participants with NAFLD (Supplemental Figure 2) and GH43_34, (β-xylosidase, α-L-arabinofuranosidase, Supplemental Figure 2; q < 0.1). This enzyme is found in the genera *Blautia (*GenBank: QBE95338.1, GenBank: ANU78801.1) and *Lachnoclostridium (GenBank: ANU49904.1)*, that were significantly enriched in the individuals with NAFLD cases (Supplemental Figure 2).

In the mediation models, plasma LBP concentrations mediated 22% (95% CI: 10–34%; *p* < 0.001) of liver fat’s effect on CRP. When we stratified the mediation models by ethnicity, plasma LBP concentrations in Latinos mediated 44% (95% CI: *p* < 0.01) of liver fat’s association with CRP. We initially considered whether LBP modified the association of liver fat with CRP within ethnicities by fitting a linear model containing the interaction of liver fat and LBP. We found no evidence for this and concluded that LBP mediates, rather than modifies, the effect of liver fat on CRP.^50^

## Discussion

In the MEC-APS, where we previously reported substantial ethnic differences in the propensity for NAFLD,^4^ our findings reported here suggest that the GM may contribute in part to the differences. We found that the microbial measures of alpha diversity, microbiome composition, and novel pathophysiologic links through gut bacterial metabolic pathways and endotoxin, were associated with NAFLD, the degree to which often differed by ethnicity. Participants with NAFLD had significantly lower alpha diversity overall and in 3 out of 5 ethnic groups, individually. No single genus was associated with NAFLD across all ethnicities, although different genera associated with production of secondary bile acids were found in common in at least three ethnicities. Carbohydrate metabolism and endotoxin-mediated inflammation were significantly higher in individuals with NAFLD. Ethnic-specific genera that ferment carbohydrates to acetate, a precursor for lipogenesis, were enriched in NAFLD subjects, suggesting common pathophysiologic links in the microbiome contribute to NAFLD.

Dysbiosis associated with decreased diversity of the microbiome starts early in simple liver steatosis and may lay the foundation for progression to inflammation and fibrosis.^51,52^ Here, we observed that not only did individuals with NAFLD, independently of their total fat mass, have a lower diversity of the gut microbiome (independent of host total fat mass) (Figure 1d), but this association was especially strong among African Americans, Whites, and Latinos (Supplemental Figures 6D and 9D, respectively). Others have shown an inverse association between alpha diversity and NAFLD.^14,53,54^

Bacterial carbohydrate metabolism that may impact lipogenesis is altered in NAFLD.^10^ Our findings suggest a pathophysiologic shift in the microbiome that supported lipogenesis in NAFLD. Increased carbohydrate metabolism was accompanied by the ethnic-specific enrichment of genera that ferment carbohydrates to acetate^55,56^ (*Blautia* in Japanese Americans, *Escherichia-Shigella* in African Americas and whites, and *Klebsiella* in whites) and an enrichment of enzymes carbohydrate metabolism by acetate producing *Blautia*, enriched with NAFLD in Japanese Americans with NAFLD (q < 0.01) (Figure 2, Supplemental Figure 2). In contrast, there was a reduction in butyrate pathways and genera (*Ruminococcus* and Lachnospiraceae;*Coprococcus*) in whites with NAFLD as others have found^9,13,57^ (Figure 3; Supplemental Table 5). Bacterially produced acetate impacts hepatic lipogenesis not only directly, as a fatty acid precursor, but indirectly as well.^58,59^ Lipoprotein lipase (LPL) is inhibited by fasting induced adipocyte factor (FIAF) that is produced in hepatocytes and when there is a shift in the gut bacteria that ferment carbohydrates to acetate, it increases lipid storage in the liver.^58–61^

13Adults with NAFLD have altered bile acid metabolism as shown in small case control studies and in larger, although ethnically homogenous, cohort studies.^54,62,63^ Imputed secondary bile acid synthesis pathways were significantly increased in all participants with NAFLD (Figure 3; Supplemental Table 5). In an ethnic-specific manner, ALT, a clinical measure of liver dysfunction, was positively associated with secondary bile acid producing bacteria and support this microbial-driven pathophysiologic link to NAFLD (Figure 2). Positive associations between ALT and certain genera have been noted in other studies.^10, 14^ Secondary bile acids have been identified as antagonists of the farnesoid X receptor (FXR), involved in lipid metabolism and glucose homeostasis (reviewed in^64^) and may lead to predisposition to liver injury and form the pathophysiological basis for clinical therapies.^65^ Therefore, a GM that produces secondary bile acids could enhance lipid accumulation in the liver through reduced liver FXR activation. The *R. gnavus* group and *Escherichia-Shigella* have members that convert primary to secondary bile acids.^66^ In our study, *R. gnavus* was positively associated with NAFLD in Latinos, Japanese Americans, and whites whereas *Escherichia-Shigella*, was significantly enriched in African Americans with NAFLD. *Lachnoclostridium* and *Blautia*, involved in the 7α-dehydroxylation of primary bile acids to secondary bile acids,^67–70^ were positively associated with NAFLD overall (Supplemental Tables 1B), and in Latinos or Japanese Americans, respectively (Figure 2). Enrichment of carbohydrate metabolizing enzymes in *Lachnoclostridium* was shown in Latinos (q = 0.03) (Figure 2, Supplemental Figure 2). Microbial metabolism of taurine and glycine involved in the deconjugation of primary bile acids with NAFLD was enriched in Native Hawaiian women^71–76^ as was *Parasutterella*. This genus has been associated with secondary bile acid metabolism and reduction in tauro-conjugated bile acids in pre-clinical models.^77^ Multiple unique genera impact bile acid metabolism and suggest that novel treatments such as FXR agonists may be altered in advanced disease across multiple ethnicities although currently this has only been tested in preclinical models.^78–80^

Endotoxin (or lipopolysaccharide, LPS) from gram negative bacteria binds to LBP and activates *toll-like receptor 4 (*TLR4) in the liver, releasing pro-inflammatory cytokines and chemokines through the NFk-B inflammation cascade and may be an important contributor to the progression from NAFLD to inflammation-based liver disease.^52,81–83^ In individuals with NAFLD, we observed enrichment of endotoxin-producing bacteria in Enterobacteriaceae, Pasteurellaceae, and Veillonellaceae (Supplemental Table 1B), although different genera were positively associated with NAFLD in different ethnic groups (Figure 2, Supplemental Table 1B). The abundance of Pasteurellaceae was a predictor of morality linked to acute-on-chronic liver failure in a longitudinal study of 42 Chinese subjects.^78^ Additionally, we showed that overall systemic inflammation (as measured by CRP) was associated with NAFLD and, among Latinos, this was potentially mediated through the LBP pathway. Other investigators could differentiate mild/moderate NAFLD from advanced fibrosis with inflammation by an increase in endotoxin-producing β-Proteobacteria, especially *Escherichia coli, Enterobacter, Aggregatibacter*, and *Klebsiella*, as we found in Japanese Americans, African Americans, and whites.^11,44^ Additionally, Sookoian et al. showed that LPS was enriched in individuals with liver steatosis in Latinos^70^ and we found *Aggregatibacter*, an endotoxin producing bacteria, was significantly more prevalent in Latinos with NAFLD (Supplemental Table 1B). *Megamonas*, a member of the Veilonellaceae that also produces endotoxin, was also significantly prevalent and positively associated with NAFLD in Native Hawaiians (Supplemental Table 1B) suggesting a novel pathway linked to systemic inflammation in NAFLD unique to this ethnic group.

Oral bacteria have been implicated in inflammation-based diseases (e.g., cardiovascular disease, T2D, colorectal cancer (CRC), and NASH).^84,85^ We observed a higher abundance *Fusobacterium, Aggregatibacter*, and *Alloprevotella* in participants presenting with NAFLD, which varied by ethnicity (Figure 2). In our study, *Alloprevotella* was significantly increased in whites with NAFLD and others have *Alloprevotella* enriched in liver biopsies in subjects with NAFLD.^70^ *Aggregatibacter* has been associated with NAFLD and altered glucose metabolism.^46,47^ *Fusobacterium* was significantly decreased in African Americans with NAFLD (Figure 2, Supplemental Figure 4). In contrast, whites with NAFLD showed a significant enrichment in *Fusobacterium* and a higher percentage of whites had *Fusobacterium* in their stool (Figure 2, Supplemental Figure 4). Fecal enrichment in stool of the oral pathogen *Fusobacterium*, has been associated with NAFLD and with inflammation and fibrosis in NASH.^12,61,86^ The clinical relevance of *Fusobacterium* may be as an alterable prognostic marker linked to prevention through changes in periodontal and oral hygiene.^87^

The GM from healthy individuals have high diversity bolstered by butyrate producing bacteria, a key energy source of gut epithelium.^88^ In our study, the butyrate-producing genera of the Lachnospiraceae and Ruminococcaceae were diverse but these patterns were unique to different ethnic groups. The *Bacteroides pectinophilus* group, which degrade complex carbohydrates found in fiber, was significantly increased in Native Hawaiians, Japanese Americans, and African Americans without NAFLD. The *Christensenellaceae R-7* group was depleted in NAFLD in African Americans but enriched in whites.^89^ Several large human population studies have found *Christensenella* were enriched in healthy weight individuals and inversely associated with NAFLD.^90,91^ Christensenellaceae and members of this consortia has also been associated with a lean phenotype in humans and mouse models of obesity, a shift in SCFA metabolism,^92^ and members of the Christensenellaceae are one of the most strongly heritable bacterial groups.^89,93^

A major strength of this study is the broad representation of ethnicity, sex, and BMI and corresponding well-curated metadata, including the state-of-the-art MRI measurement of liver fat used both to determine NAFLD status and to analyze as a continuous variable, comprehensive bacterial profiling, and metagenomic analysis. While our diverse study population supports the generalizability of the findings, given the older age of the individuals, the results may not be generalizable to younger individuals. We did not include medication use in our models which may independently alter NAFLD. Although some of our subjects with NAFLD may have been using metformin to control T2D,^94^ a recent meta-analysis suggests that while weight and glucose control were improved with metformin, it did not substantially impact liver disease, studies suggest that metformin does not alter NAFLD status.^95^ Additionally, subjects were excluded from the MEC APS if they had cancer and other bowel diseases. Therefore, subjects using chemotherapy drugs shown to impact NAFLD would have been excluded and reduce the probability of this bias.^95^ In this study, we did not assess liver fibrosis that may have different associations with GM than steatosis. Although our results are suggestive of several candidate bacteria and pathways for NAFLD etiology, our single timepoint analysis does not provide strong causal inferences that the associated GM traits affect temporal changes in liver fat. Additionally, we had smaller sample sizes when stratified by ethnicity. Longitudinal studies are needed, especially in different ethnic groups, to establish the role of the microbiome in the development of fatty liver and the transition to more inflammatory and fibrotic liver diseases. Further, extending beyond our GM analysis, the application of metagenomic sequencing approaches that allow genomic reconstruction will identify different species and strains that may impact NAFLD.

We have observed that aspects of the GM composition and metabolism are associated with NAFLD, overall and in ethnic-specific manners among generally healthy older adults. Additionally, systemic inflammation may be mediated in part by the microbiome and varies by ethnicity. Microbial-associated mechanisms may provide insight into the development of NAFLD. Once replicated in other studies, ethnic-specific microbial composition and pathophysiologic pathways can provide the basis for targeted therapies, such as narrow spectrum antibiotics,^96^ diet,^6,97^ fecal transplants^98,99^ or phage therapies,^100^ for future clinical treatment specific to the microbiome. Microbiome-mediated pathways may provide an actionable ethnic-specific target to reduce inflammation and reduce the transition from simple steatosis to advanced disease.

## Supplementary Material

Supplemental Material

## Data Availability

Sequencing files have been submitted to the SRA for public access (PRJNA629344).
