# Supplementary material for: Associations of the gut microbiome with hepatic adiposity in the Multiethnic Cohort Adiposity Phenotype Study
Source: Gut Microbes. 2021 Sep 7;13(1):1965463. doi: 10.1080/19490976.2021.1965463 (PMC8425768; doi:10.1080/19490976.2021.1965463)
Supplement: Supplemental Material [file KGMI_A_1965463_SM0075.zip › Supplementary information/GM NAFLD Supplemental Figures.docx]

**Supplement material**

**Supplemental Figure 1.**  Phyla-forest plots of the NAFLD coefficient from each beta-binomial model, on the log-odds scale. * denotes an association with a BH-adjusted q < 0.05. Red=positive association Blue=negative association.


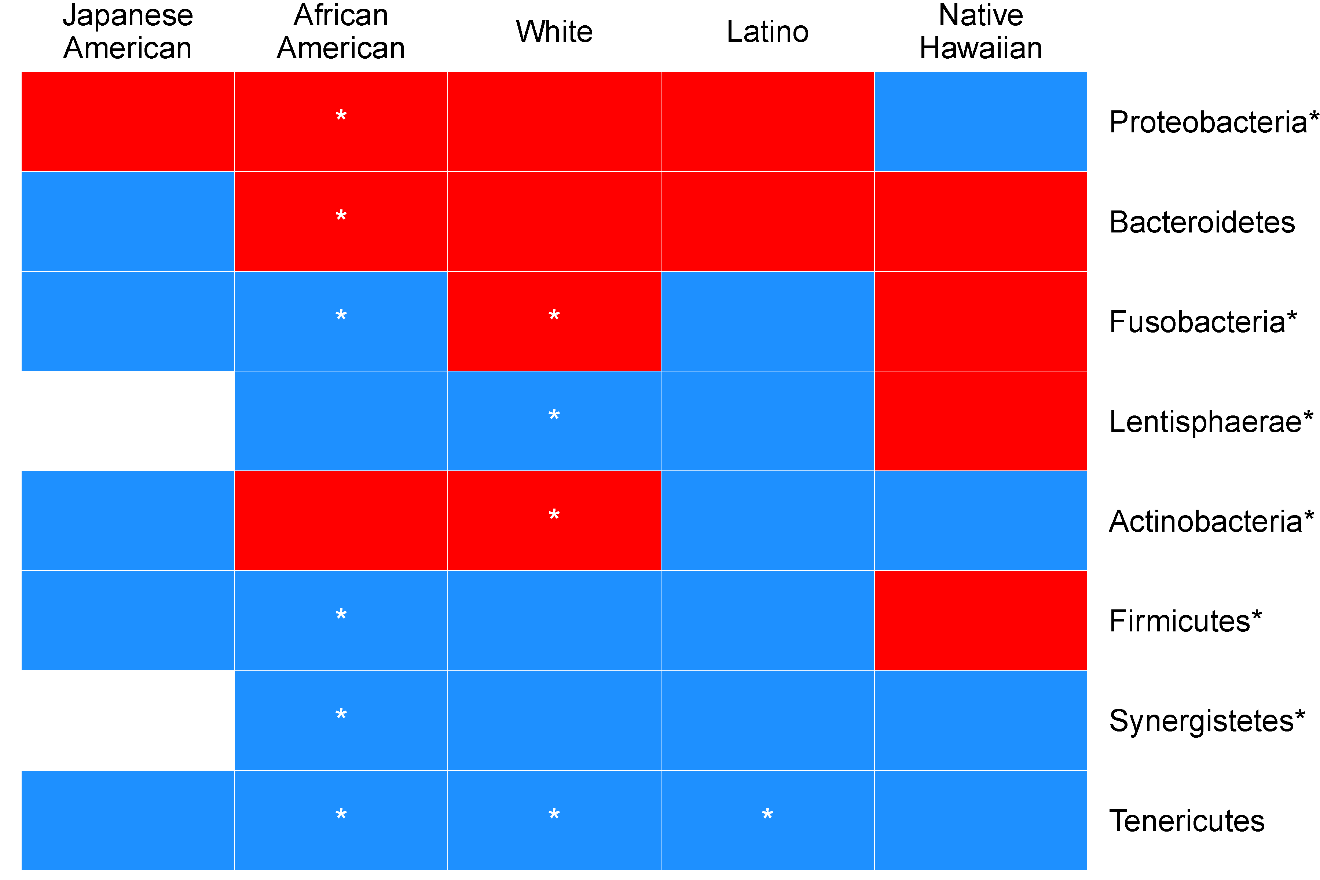


**Supplemental Figure 2.** The relative abundance of the functional genes associated with microbial metabolism in NAFLD. Most notably, carbohydrate metabolism and GH43_34 (Glycoside hydrolase group 43_34 in the Cazyme database* includes β-xylosidase, α-L-arabinofuranosidase)) were enriched in participants with NAFLD (liver fat >5.5%). This enzyme is found in the genera *Blautia (*GenBank: QBE95338.1, GenBank: ANU78801.1) and *Lachnoclostridium (GenBank: ANU49904.1)*, genera that were also significantly enriched in the participants with NAFLD. Branched chain amino acids and butyrate production were significantly lower in participants with NAFLD. Details of the genes in the pathways and metagenomic approach is described in the methods.


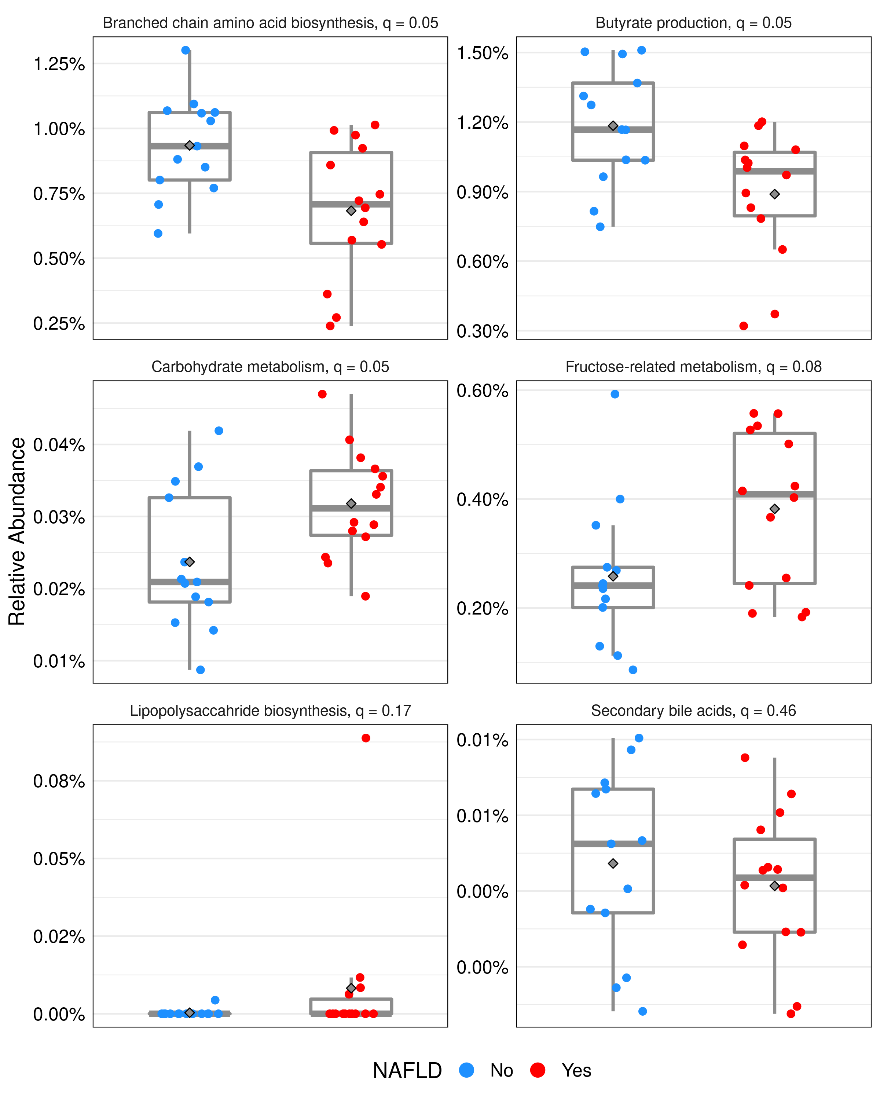

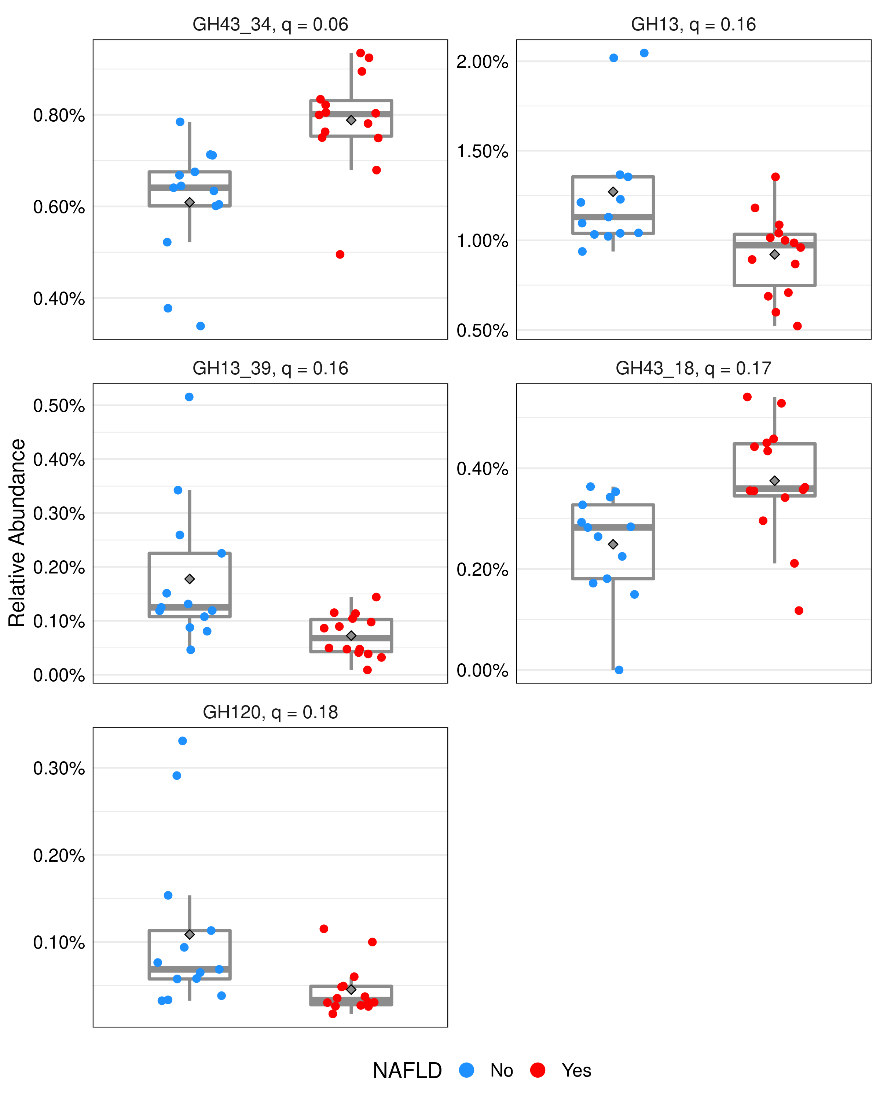


*Baris E. Suzek, Yuqi Wang, Hongzhan Huang, Peter B. McGarvey, Cathy H. Wu, the UniProt Consortium, UniRef clusters: a comprehensive and scalable alternative for improving sequence similarity searches, Bioinformatics, Volume 31, Issue 6, 15 March 2015, Pages 926–932, <https://doi.org/10.1093/bioinformatics/btu739>

**Supplemental Figure 3.** The association of BMI with % liver fat across ethnicity and sex.


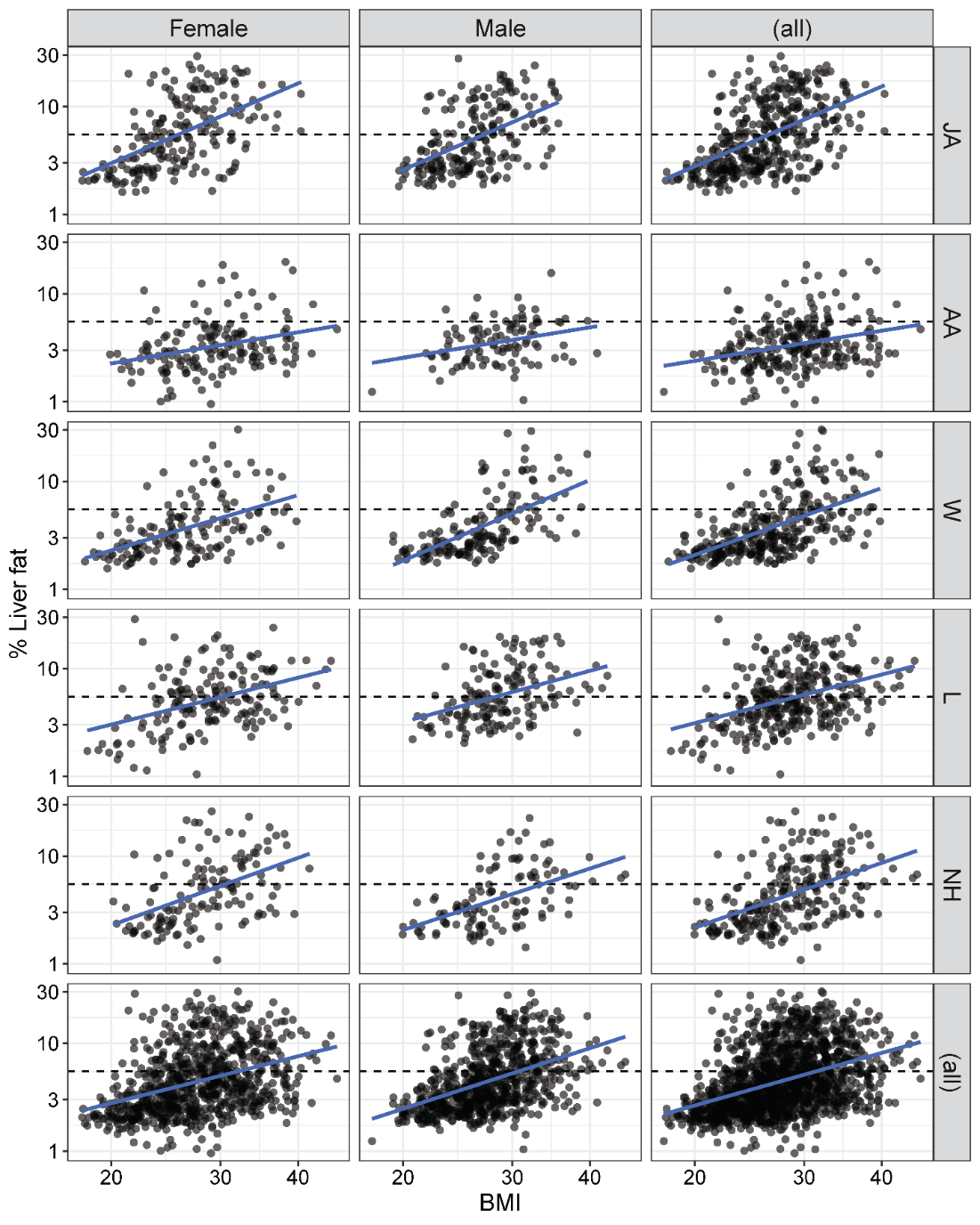


Abbreviations: JA, Japanese Americans; AA, African Americans; W, white; L, Latino; NH, Native Hawaiian.

**Supplemental Figure 4. T**he prevalence and abundance of Fusobacterium across all racial/ethnic groups and the prevalence by low (≤5.5%) and high liverfat (>5.5%) within racial/ethnic groups. The prevalence of Fusobacteria across ethnicities is Japanese American (44%) > Native Hawaiian (39%) >white(35%)>African American (19%)>Latino (16%). Within the high liverfat group (>5.5%), the prevalence is Japanese American (50%)>white (48%), Native Hawaiian (39%), Latino (19%), and African American (8%).


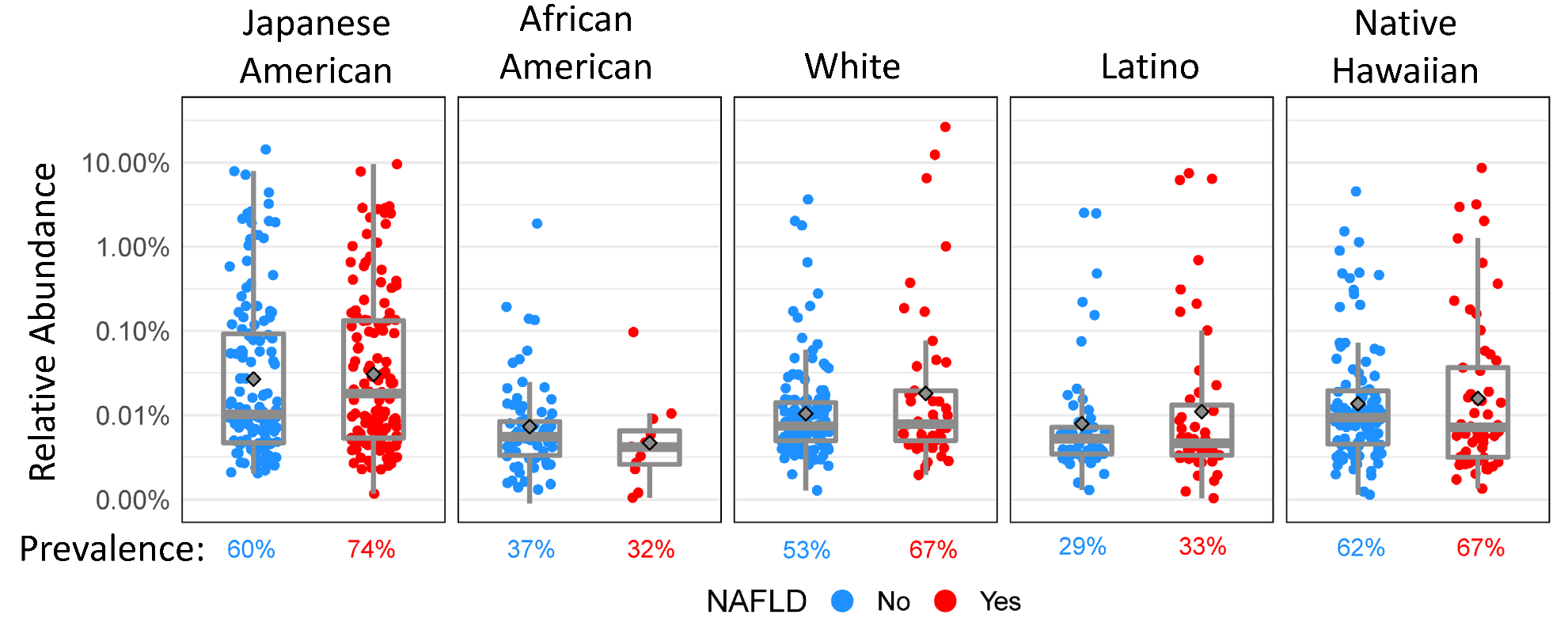


**Supplemental Figures 5-9 have the same format for each, Japanese Americans (Supplemental Figure 5), African American (Supplemental Figure 6), white (Supplemental Figure 7), Latino (Supplemental Figure 8), and Native Hawaiian (Supplemental Figure 9).** Overview of the microbiome in NAFLD in the MEC-APS study: A.) distribution of participants with and without NAFLD, B.) variation in the microbiome between participants with and without NAFLD illustrated by a PCOA of weighted unifrac metric, C.) vector overlay of the demographic anthropometric health diet and lifestyle variables, D.) alpha diversity in participants with and without NAFLD by ethnicity, E.)variation in the microbiome explained by the demographic anthropometric, health, diet, and lifestyle variables using perMANOVA. Analysis, and F) Forest plots of beta coefficients by genera.


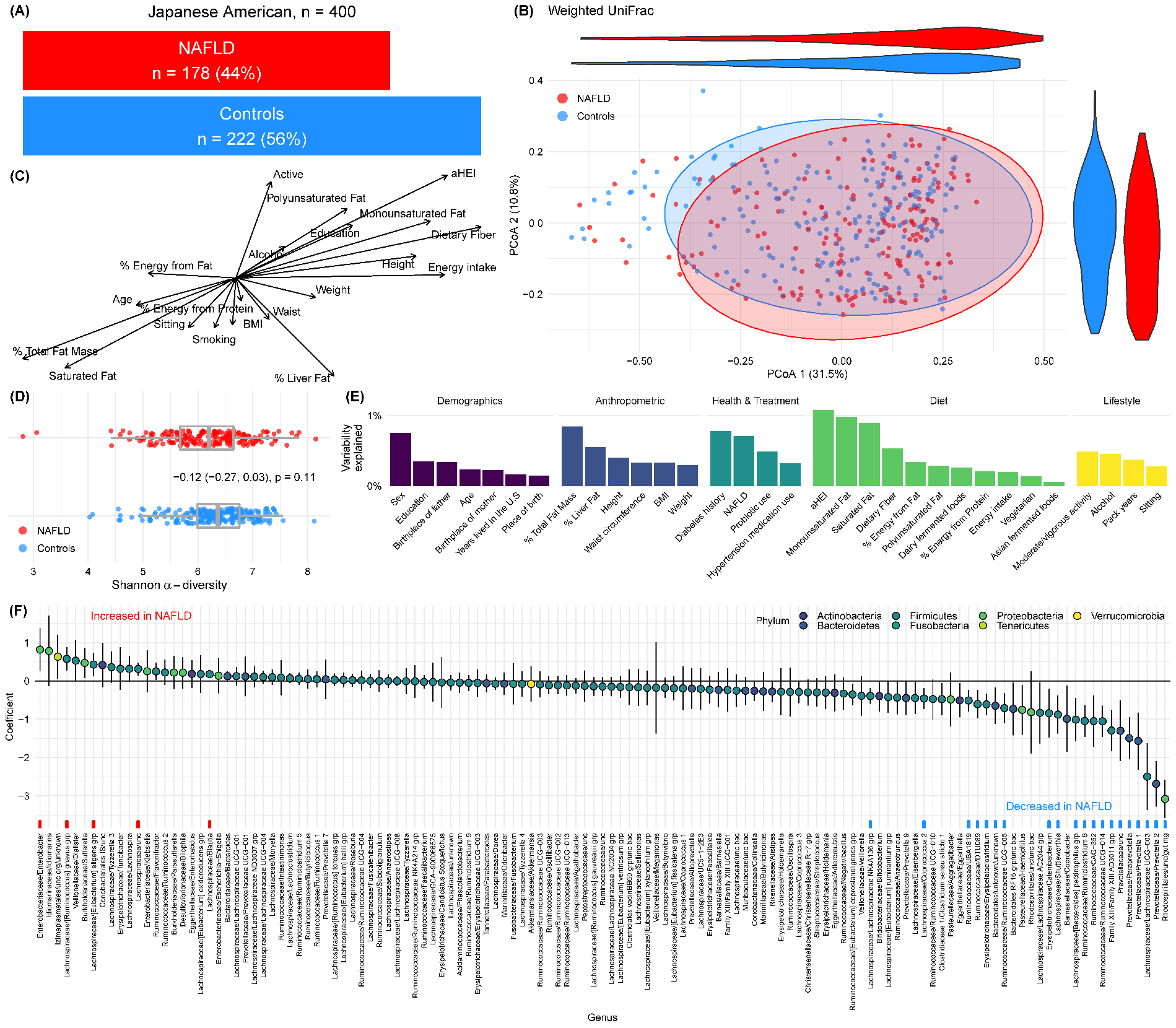
**Supplemental Figure 5 - Japanese American**


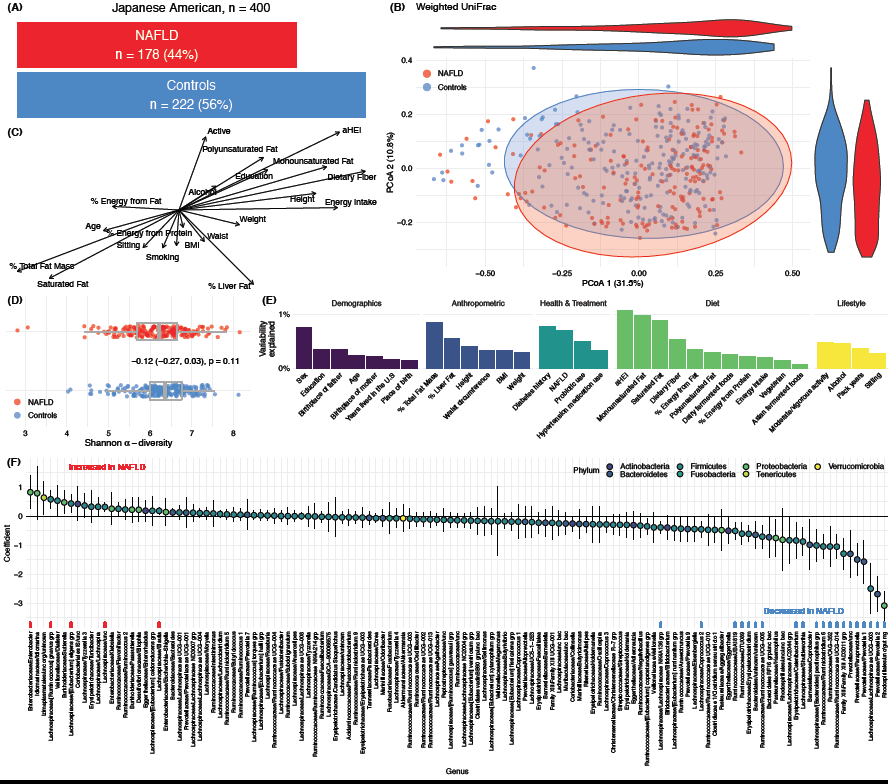


SFigure 7 African Americans

**Supplemental Figure 6 - African American**

**
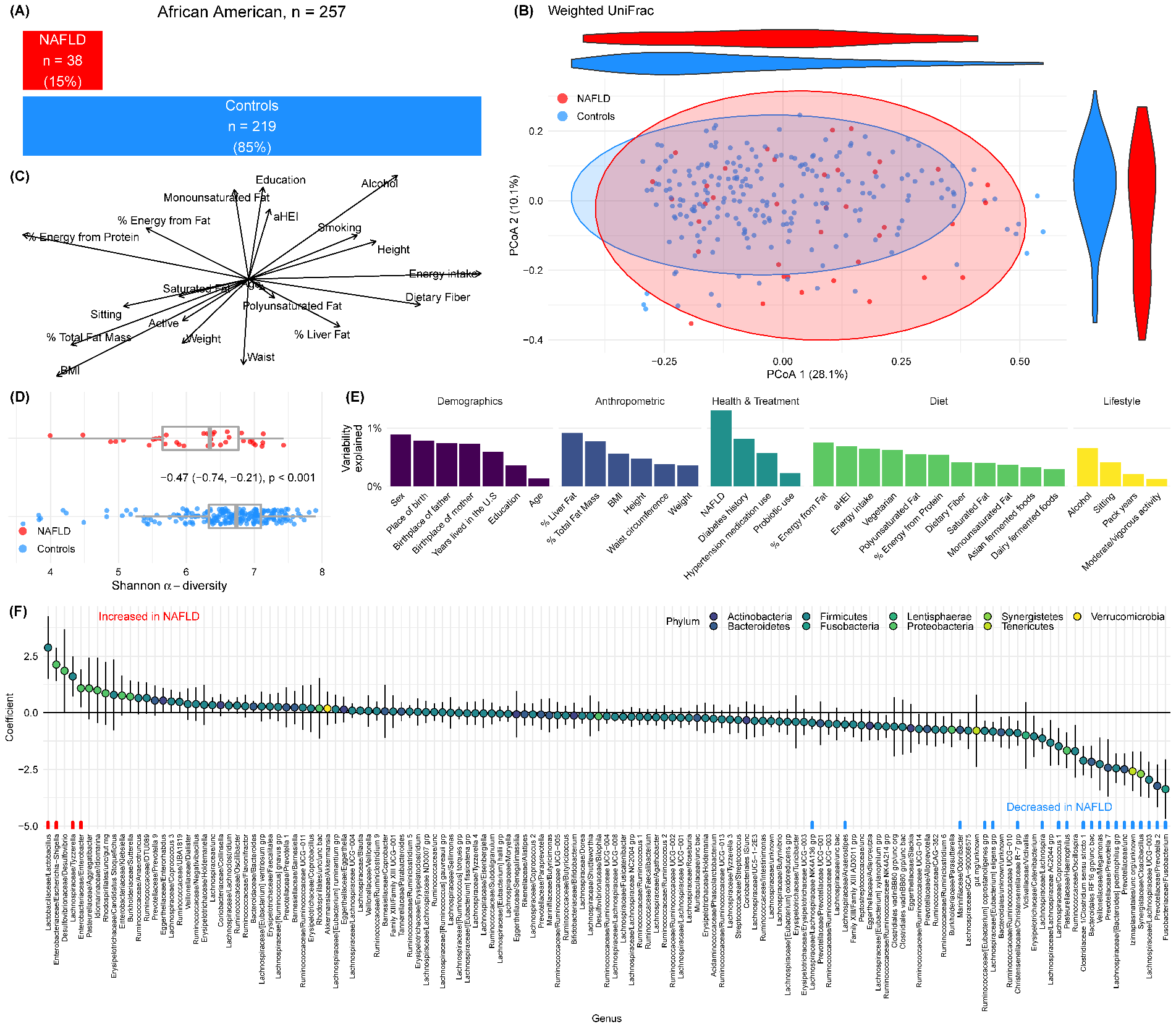
**

SFigure 8. White

SFigure 9

**SFigure 10 Native Hawaiaan**

**Supplemental Figure 7 - White**

**
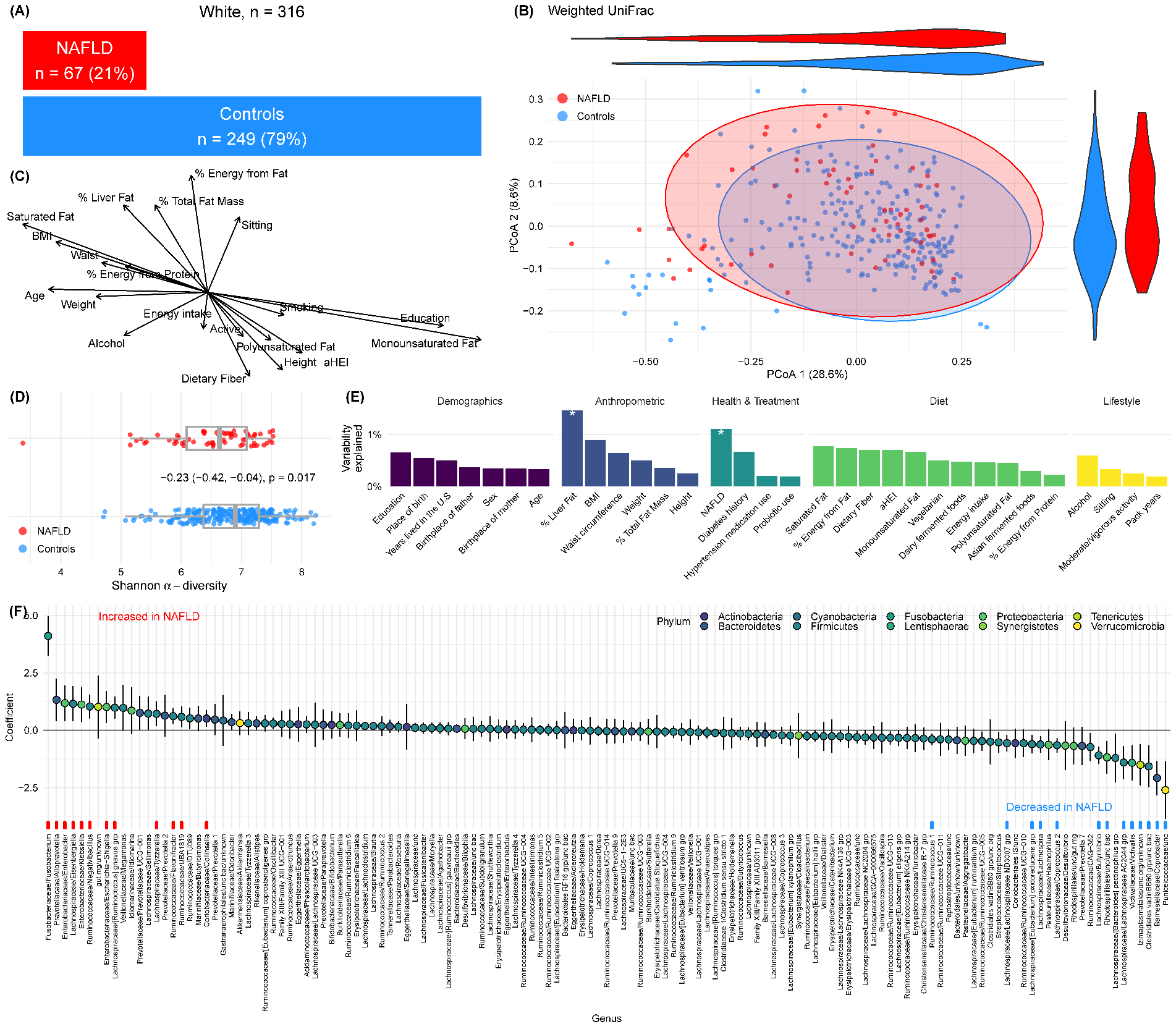
**

**Supplemental Figure 8 - Latino**

**
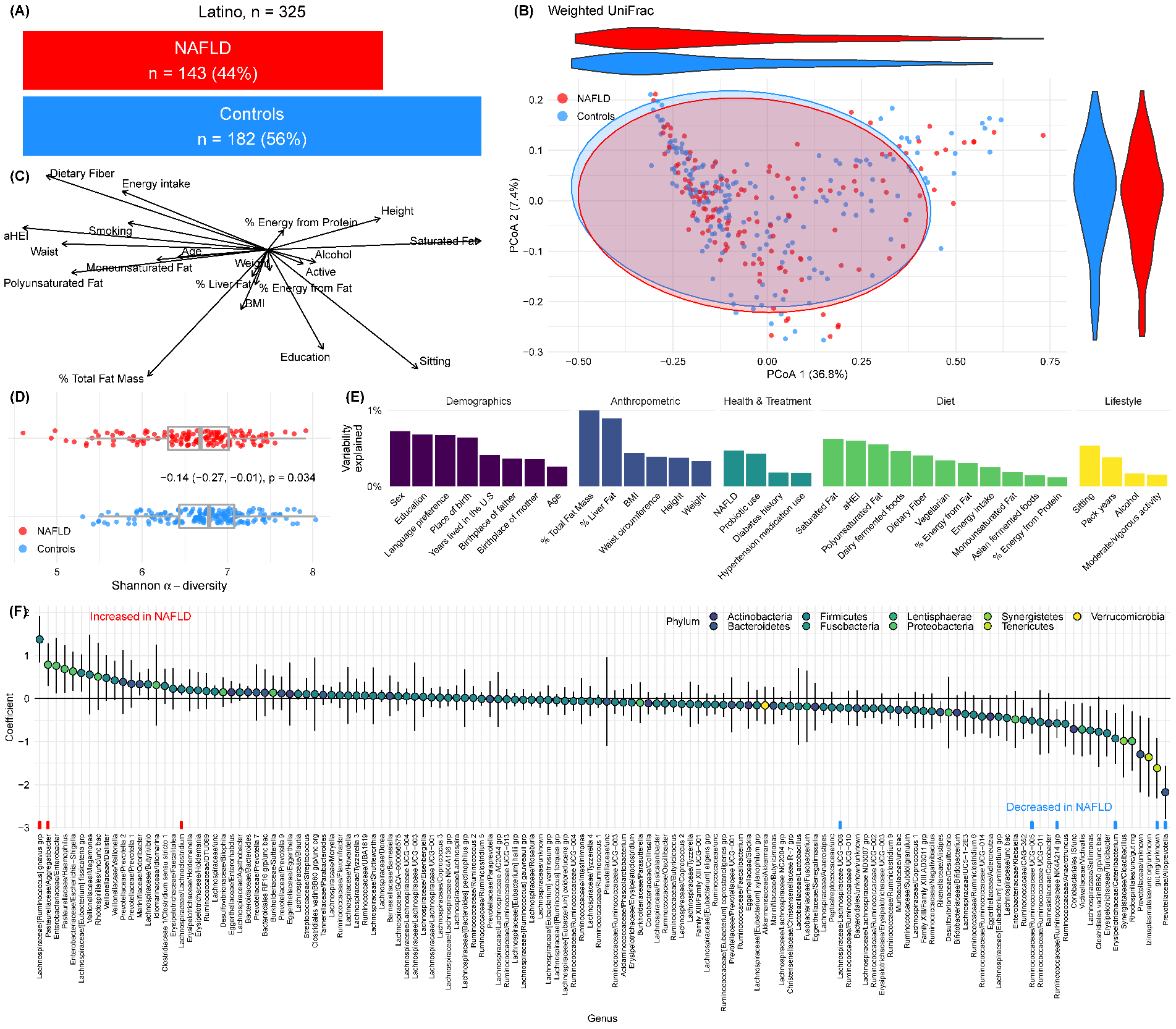
**

**Supplemental Figure 9 - Native Hawaian**

**
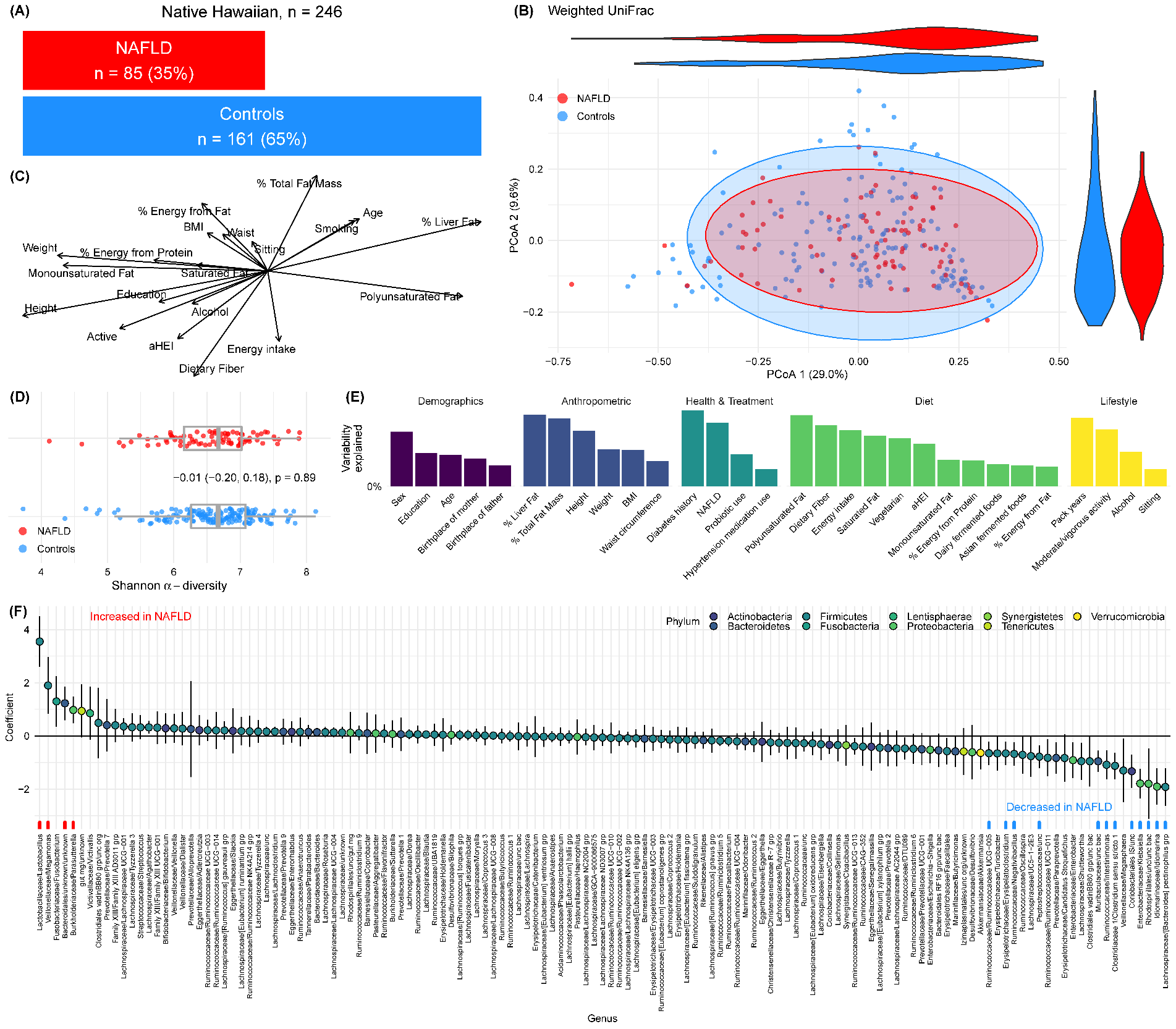
**
